# Oral Juvenile Xanthogranuloma: a case report of gingival hyperplasia and osteolysis in male adult patient

**DOI:** 10.1186/s12903-022-02643-y

**Published:** 2022-12-18

**Authors:** Long Chen, Lin Feng, Lingling E

**Affiliations:** 1 Department of Stomatology, People’s Hospital of Mancheng District, Baoding, 072150 Hebei China; 2 Department of Oral and Maxillofacial Surgery, First Medical Center of Chinese PLA General Hospital, Beijing, 100853 China; 3 Institute of Stomatology and Oral Maxilla Facial Key Laboratory, First Medical Center of Chinese PLA General Hospital, Beijing, 100853 China

**Keywords:** Juvenile Xanthogranuloma, Non-Langerhans cell histiocytosis, Case report

## Abstract

**Background:**

Juvenile Xanthogranuloma (JXG) is a non-hereditary, self-limiting disease which is usually presented in infancy or early childhood and in males over females.

**Case presentation:**

We report a rare case of oral Juvenile Xanthogranuloma with recurrent progressive gingival hyperplasia and concomitant presentation of osteolysis in a 21-year-old adult male with no significant medical history. Patient presented with generalized gingival hyperplasia, osteolysis of the maxilla and mandible, and a round, firm, nodular mass with clear circumference on the left shoulder. Results of gingival tissue biopsy, karyotype, bone marrow biopsy and immunohistochemistry were suggestive of a diagnosis of Juvenile Xanthogranuloma with no association to hematologic malignancy. Unfortunately, patient declined treatment and elected to be transferred back to local hospital for future evaluation.

**Conclusions:**

Juvenile Xanthogranuloma in adults can have atypical manifestations including generalized gingival hyperplasia and osteolysis of the maxilla and mandible. It should be differentiated between Langerhans cell histiocytosis, Papillon–Lefevre Syndrome, and Pyogenic Granulomas. Despite uncommon incidence, it should be included in differential diagnoses in cases of similar clinical presentations.

## Background

Juvenile Xanthogranuloma (JXG) is a non-hereditary, self-limiting disease which is usually presented in infancy or early childhood and in males over females. It is a non-Langerhans cell histiocytosis with unknown etiology, though it has commonly been characterized by proliferation of monocytes as a granulomatous response to physical, chemical or infectious stimuli [1, 2]. The typical clinical presentation consists of solitary or multiple yellow-orange-brown firm papules or nodules. The most common locations are the face, neck, and upper torso. Other bone, visceral and or CNS involvement is uncommon. Moreover, oral JXG is especially rare in adults [3]. Herein, to our best knowledge, we report for the first time a case of oral JXG with the concomitant presentation of progressive gingival hyperplasia and osteolysis.

## Case presentation

A 21-year old male patient with no significant medical history was referred to our dental outpatient clinic in June 2020 with the chief complaint of dysphagia due to teeth mobility and sleep disturbance for 4 months. Patient has no significant medical condition and family history. The patient was previously seen for generalized gingival edema and hyperplasia in 2017 at local hospital. He was diagnosed with “pyogenic granuloma” and underwent a gingivectomy in 2018, during when surgical excision of the expanding nodular masses on both buccal and lingual surfaces of #3–#5, #7–#9, #12–13, #19–#21 and #28–#30 was conducted. Pathology results were equivocal.

**Fig. 1:**
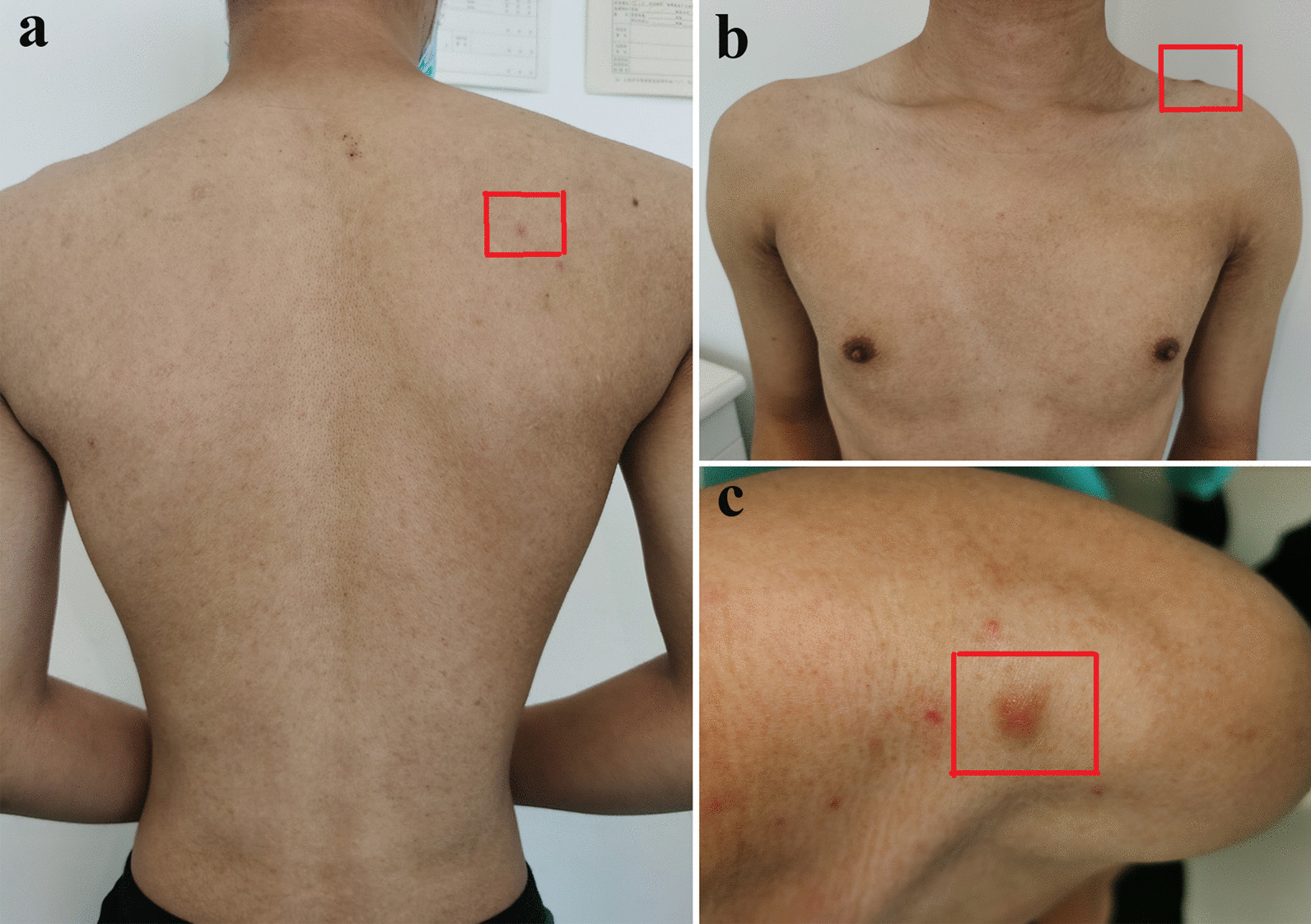
The physical examination of patient. Lesions in the back area (**a**). Lesions of the left shoulder (**b**). A round, 2.5 cm × 1.5 cm nodular mass with well-defined demarcations on the left shoulder (**c**). Red boxes indicated areas of lesion

**Fig. 2:**
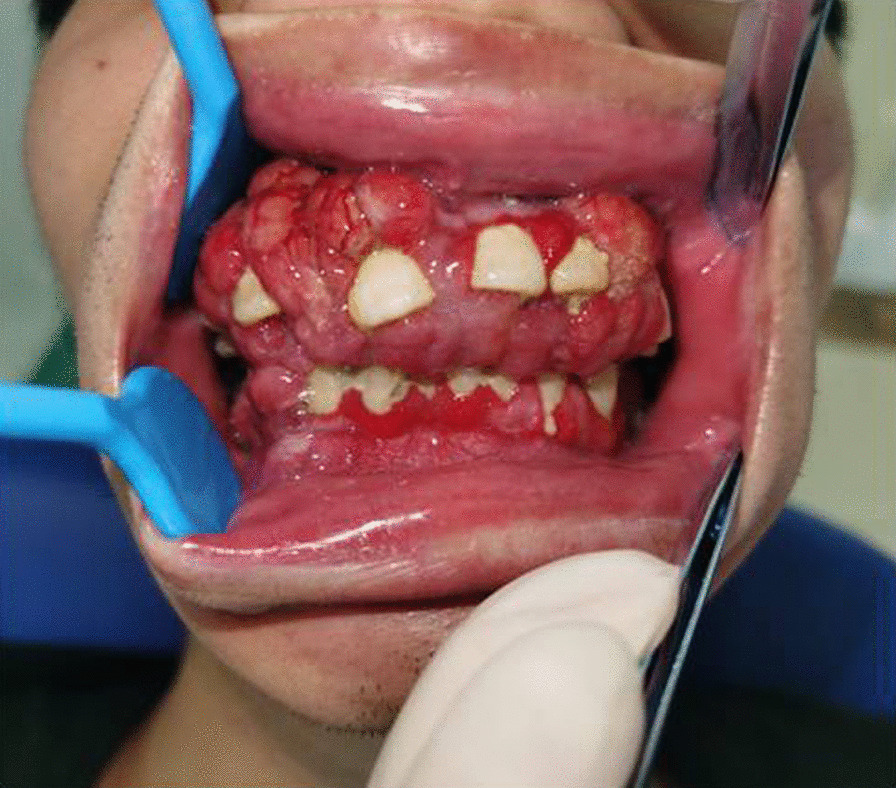
Intraoral examination revealed generalized edematous and erythematous gingiva associated with non-purulent periodontal pockets, tooth mobility, displacement was visualized on inspection

**Fig. 3:**
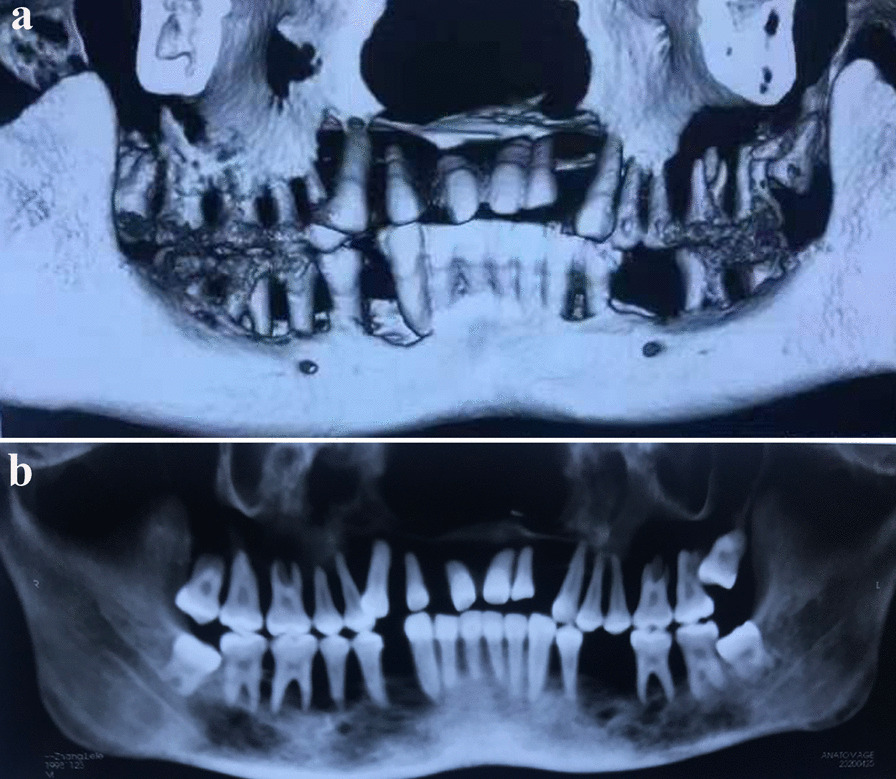
The imaging examination of the patient's jaw bone. Muiltiple ill-defined multilocular, amorphous-shaped radiolucencies in both maxilla and mandible were identified in Panomeric film (**a**). Radiographic bone loss was noticed, that bone level was seen to extend beyond the middle third of the root. The inferior border of the mandible showed punched-out lesions, partially surrounded by areas of periosteal reaction (**b**)

In April 2020, the patient presented again with gingival hyperplasia, tooth mobility and diminished ability to chew and was referred to our clinic. Clinical presentations include generalized gingival hyperplasia and osteolysis of the maxilla and mandible. Extraoral examination showed symmetrical appearance with no obvious lymphadenopathy. Intraoral examination indicated generalized dark red gingival enlargement predominantly in palatal and lingual regions, with partial extension to incisal edge and occlusal surface of the teeth. Nodular masses were located at both labial and palatal surface from regions of #6–#11 and #18–#21 with localized surface erosion. Physical examination revealed lesions on the patient's back and shoulder; a round, firm, well-demarcated 2.5 cm × 1.5 cm nodular mass was identified on the left shoulder of the patient, yet he declined a tissue biopsy at the location (Fig. 1a–c). The patient also presented with bleeding gingiva upon palpation, non-purulent periodontal pocket, tooth mobility and displacement with a “floating in air” phenomenon (Fig. 2). Radiology indicated ill-defined multilocular, amorphous-shaped radiolucencies in both maxilla and mandible. There was erosion of lamina dura with generalized tooth displacement, and radiographic bone level was seen to extend beyond the middle third of the root. The inferior border of the mandible showed punched-out lesions, partially surrounded by areas of periosteal reaction (Fig. 3a, b).

**Fig. 4:**
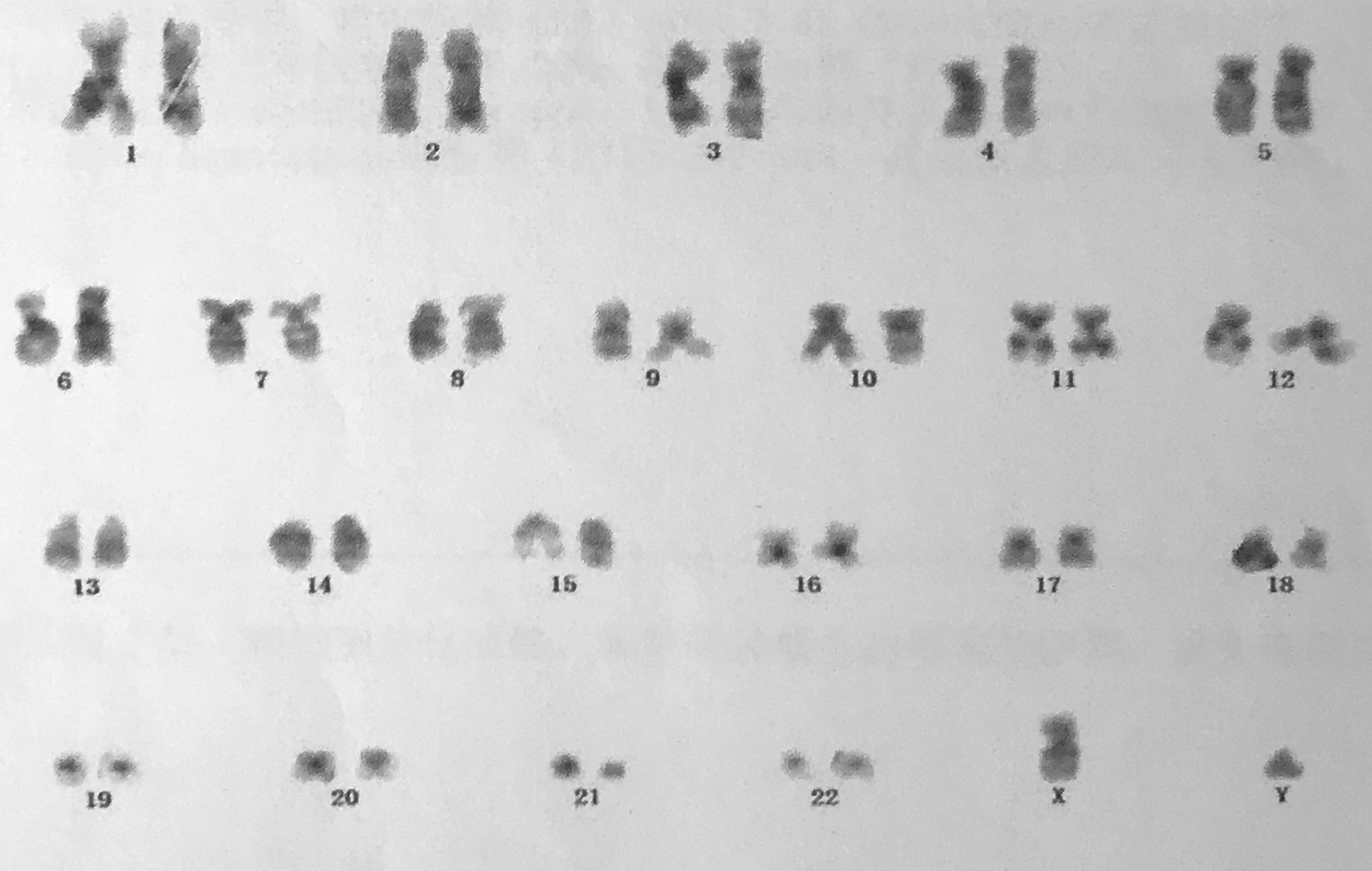
Karyotype reported normal chromosomes

**Fig. 5:**
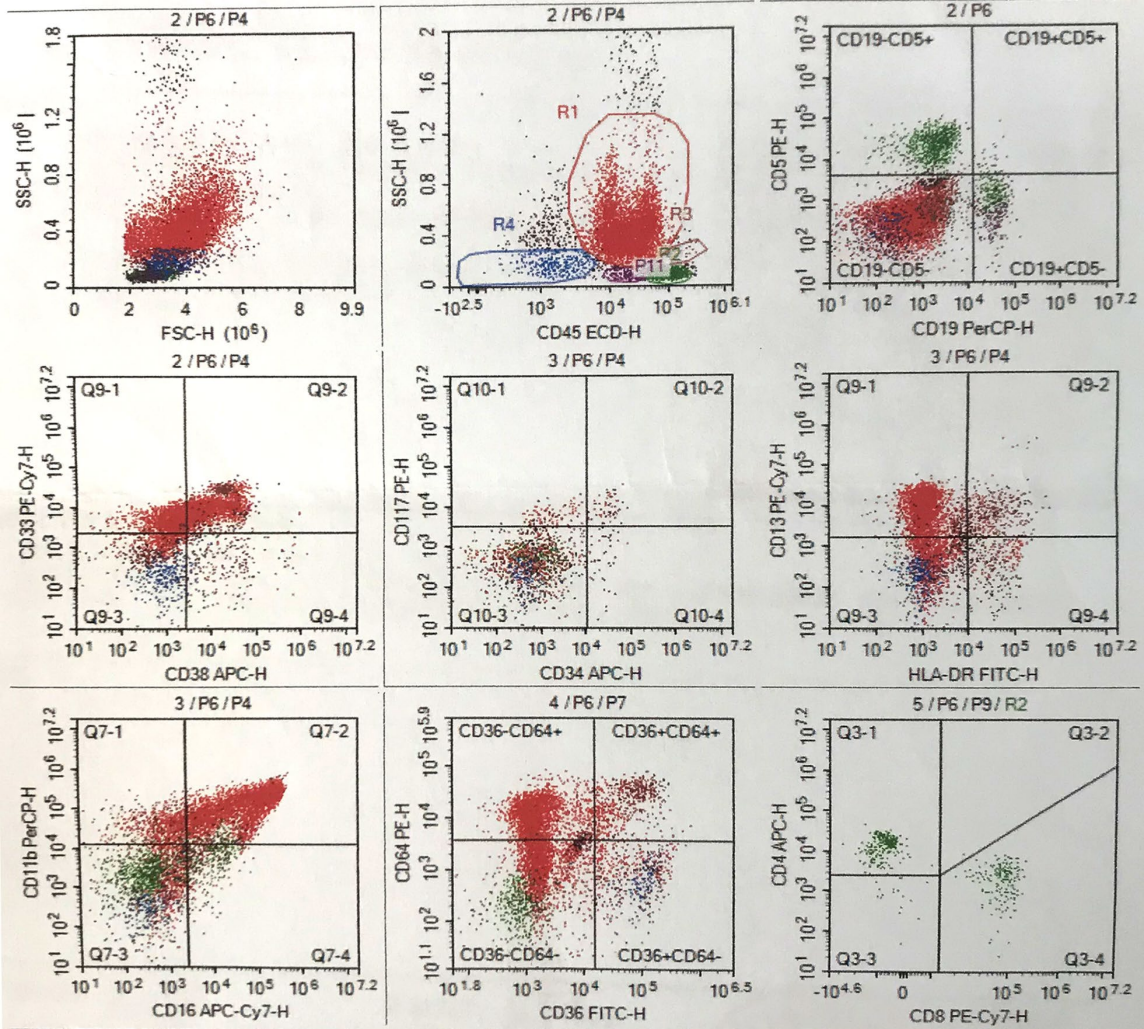
Bone marrow biopsy specimens revealed mild deficiency of granulocytes and erythrocytes proliferation, no special immunophenotype was observed. Granulocyte group (R1) accounted for 68.14%, CD16/CD11b expression curve suggested that the proportion of cells in each stage of the granulocyte was roughly normal, and CD34^+^CD117^+^ accounted for about 1.22% of nuclear cells; T cells dominated the lymphocyte population (R2), which accounted for 15.49%. CD4/CD8 ratio was normal in CD3^+^ cells, and Kappa and lambda restricted expression was not observed in CD19^+^CD20^+^ cells; monocyte population (R3) accounted for 2.32%; Nucleated red cell population (R4) accounted for 3.65%; Hematogone cell population (P11) accounted for 2.68%

**Fig. 6:**
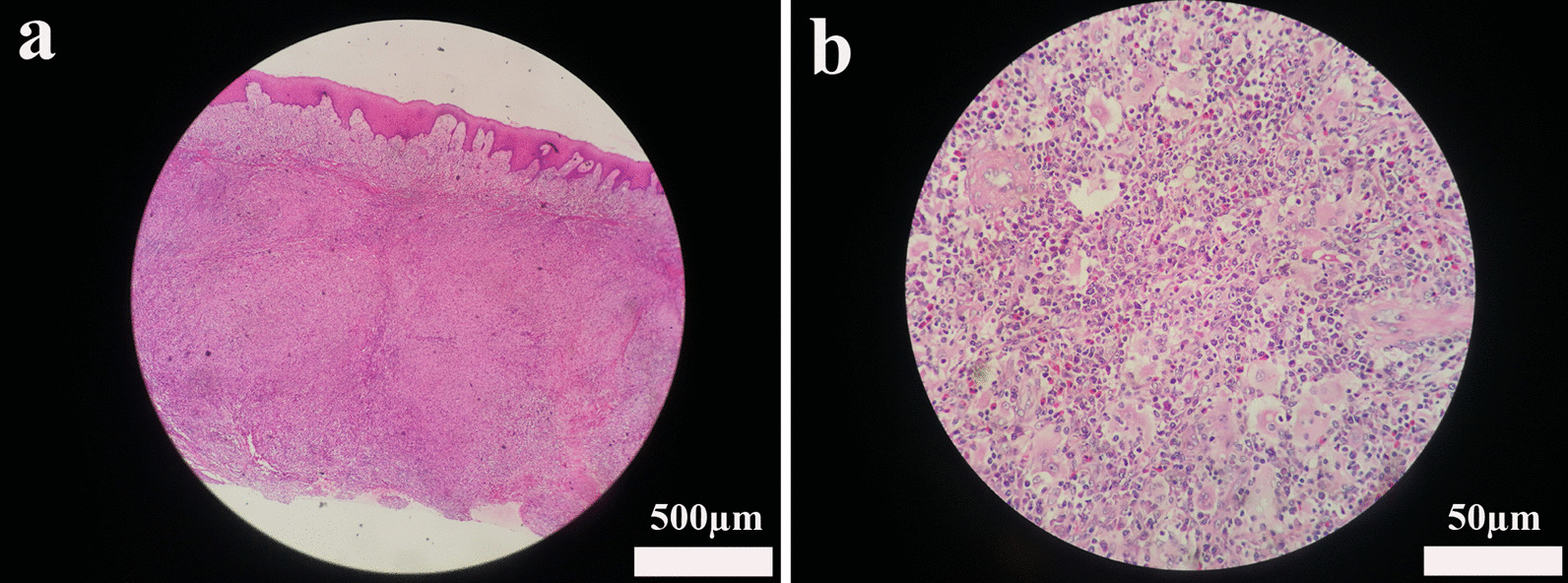
Histological observation of the mandibular hyperplastic gingival tissue. Low magnification observation of hematoxylin eosin staining (**a**) (scale bar = 500 µm). Under high magnification observation, colonies of histiocytes in lamina propria were identified, together with multinucleated giant cells, and small amounts of lymphocytes, plasma cells, eosinophils, and Touton giant cells; there were few areas of calcifications (**b**) (scale bar = 50 µm). The arrows indicated Touton giant cells

**Fig. 7:**
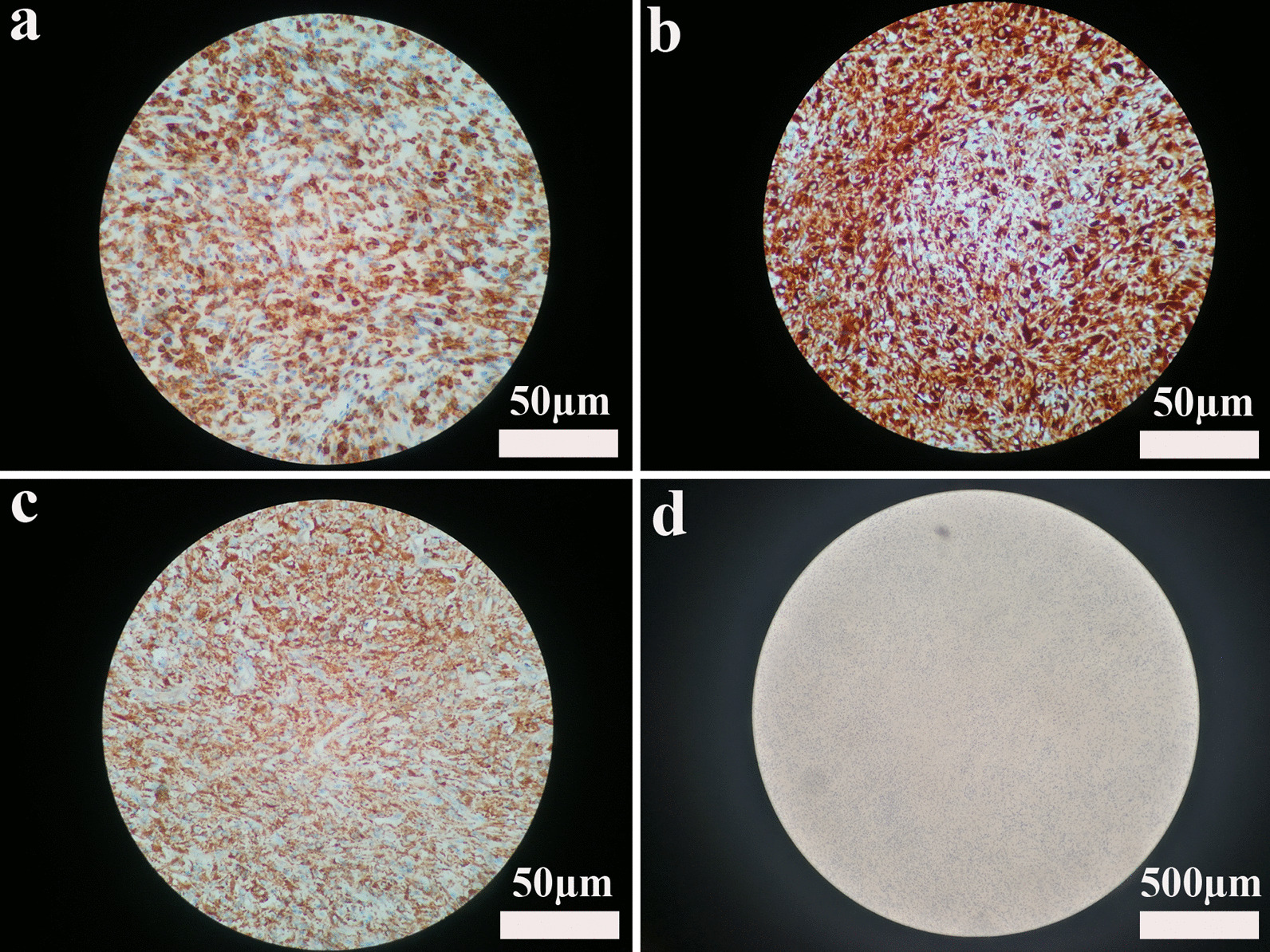
Immunohistochemical staining of the mandibular hyperplastic gingival tissue. The mandibular hyperplastic gingival tissue positively expressed LCA (**a**), Vimentin (**b**), and CD68 (**c**) (scale bar = 50 µm), negatively expressed CD1α (S-100, CD35, and CD21) (d) (scale bar = 500 µm)

Karyotype reported normal chromosomes (Fig. 4). In the cd45-SSC scattered plot, granulocyte group (R1) accounted for 68.14%, CD16/CD11b expression curve suggested that the proportion of cells in each stage of the granulocyte was roughly normal, and CD34^+^CD117^+^ accounted for about 1.22% of nuclear cells; T cells dominated the lymphocyte population (R2), which accounted for 15.49%. CD4/CD8 ratio was normal in CD3^+^ cells, and Kappa and lambda restricted expression was not observed in CD19^+^CD20^+^ cells; monocyte population (R3) accounted for 2.32%; Nucleated red cell population (R4) accounted for 3.65%; Hematogone cell population (P11) accounted for 2.68%. These results suggested mild granulocytic deficiency, erythrocyte proliferation, and no specific immunophenotype in bone marrow biopsy specimens (Fig. 5). With informed consent, a tissue biopsy was conducted by excising mandibular hyperplastic gingival tissue. Hematoxylin eosin staining showed that there were colonies of histiocytes, multinucleated giant cells and a few lymphocytes, plasma cells, eosinophils and Touton giant cells in the lamina propria; there were few areas of calcifications (Fig. 6a, b). Immunohistochemical staining reported: Langerin (−), LCA (+), CD35 (−), CD21 (−), S-100 (−), Myo-D1 (−), CD138 (−), CD34 (−), Myogenin (−), CD 38 (−), SMA (−), Vimentin (+), CK (−), CD1a (−), CD68 (+) (Fig. 7a–d).

The above findings were suggestive of a diagnosis of Juvenile Xanthogranuloma. Topical and systemic corticosteroids were recommended to the patient, with proposed treatment plan of surgical excision and removable prosthesis. However, patient declined treatment at our clinic due to financial reasons and elected to be transferred back to local hospital for future medical intervention.

## Discussion and conclusions

Reported and described by Dr. Adamson in 1905, JXG was initially addressed as congenital xanthoma multiplex [4], it was given its current name in 1954, while Gartmann and Tritsch [5], in 1963, reported the occurrence of cutaneous xanthogranuloma in adulthood. As xanthogranuloma classically present as yellow papules or nodules spreading out over head, neck and upper body, oral JXG was considered a rare finding. Sporadic cases showed that oral JXG presented with non-recurrent lesions on the gingiva, alveolar mucosa, tongue, and intra-masseter muscles. It is also interesting to note that none of the oral JXG patients had concurrent skin lesions, nor have they reported any osteolysis and lympho-proliferations [6]. While the previous cases reported solitary lesions ranging from papular size to giant size, a generalized pattern was exhibited by our patient. In addition, recurrent manifestations, seen in our patient, have not been reported in previous literature despite we were unable to follow up with the patient for medical interventions. An association to hematologic malignancy such as large B-cell lymphoma or B/T cell leukemia and JXG in adulthood has also been proposed [7], though in our case only mild proliferation of granulocytes and erythrocytes was reported.

Microscopically, JXG is often compared to Langerhans cell histiocytosis (LCH), definitive diagnosis often requires histological examination of the tissue. Histopathological observations for JXP usually reveal an initial proliferation of lymphohistiocytes in the dermis [8]. This is followed by the garland-like Touton giant cells exhibiting amorphous and eosinophilic cytoplasm surrounded by foam cells. Lastly, fibrohistiocytic cells can be observed in a later stage. Immunohistochemistry (IHC) usually reveals positive CD68, CD1a staining, with negative S100 staining. LCH, in contrast, demonstrates conflicting IHC results, as it usually leads to epidermotropic infiltration that is absent in JXG. Clinically, LCH is also characterized by temporal bone lesion, exophthalmos, and diabetes insipidus. In our patient, the immunohistochemical staining indicated positive CD68 and negative CD1α, S-100, CD35, and CD21, while Touton giant cells were also observed (Fig. 6b). This is consistent with the results of previous case reports of cutaneous JXG and thus ruling out LCH. Other differentials include Papillon-Lefevre Syndrome (PLS), which also manifest with periodontal destruction and gingival hyperplasia. While the patient did not present with keratosis at the palmoplantar or knee region associated with PLS, it might be necessary to conduct molecular genetic testing to exclude the possibility of JXG-PLS.

- Some common characteristics can be found between JXG and Pyogenic Granulomas (PG) that both may present as growing lesions at gingiva, buccal mucosa, lips and tongue. PG lesions are also usually red or pink in color, with associated loss of bone. Yet PG is more commonly seen in females in their second decade of life, partly due to hormonal changes [9]. JXG can be differentiated from PG with histopathological observations, where the latter usually has a heavy vascular component with proliferation of endothelial cells.

In spite of elusive etiology, cutaneous JXG is often self-limiting, yet oral JXG often requires surgical removal. Oral JXG was shown to have low recurrence rate after surgery as long as the JXG had no systemic involvement [10, 11]. First-line medications for JXG include corticosteroids, vincristine and methotrexate, etc., and the treatment regime is still undetermined for recurrent oral JXG due to little evidence. For the current case, prognosis remains questionable due to the presence of osteolysis, and viability of post-operative bone grafts still requires further evaluation.

JXG in adults can be atypical and present as generalized gingival hyperplasia and osteolysis of the maxilla and mandible, and an evaluation of lymphoproliferative disorders could indicate mildly deficient bone marrow proliferation. Therefore, in cases with similar clinical presentations, JXG must be considered in the differential diagnosis.

## Data Availability

Not application.

## Abbreviations

JXG: Juvenile Xanthogranuloma
LCH: Langerhans cell histiocytosis
IHC: Immunohistochemistry
PLS: Papillon–Lefevre Syndrome
PG: Pyogenic Granulomas
